# Letter from Italy

**Published:** 1885-01

**Authors:** A. Lagorio

**Affiliations:** Chiavari


LETTER FROM ITALY.

*Messrs. Editors:*—During the past six months our sunny and beautiful land has suffered a severe epidemic of cholera, of secret nostrums, and of pamphlets. The latter still continues. I have before me a pamphlet four by six inches in size, printed in large type, entitled, "The Cholera—Its Origin—Development—and Remedies." Its author, Leonard L., has had the skill to cover the entire subject in twelve pages, including the frontispiece!

Ever since I read over and over again this production of Leonard's brain, I have been wondering in my mind if Koch and the other scientists really know anything about cholera.

Leonard tells us that for a long time he has been hesitating whether he should publish his ideas on a subject so grave and important as that of the cholera, because he thought it was almost time that the most celebrated physicians of the world should know how to solve the difficult problem. But, so far, no one has, he says, described the cause or the remedy for this fatal disease; and, therefore, its general extension, instead of diminution, tending to complicate the public calamity with absurd and inhuman precautions, he has thus not only the courage, but feels also obliged to face the criticism and the derision of the great majority, accustomed to bow blindly only to the idols of modern celebrities, most of them risen, not by their merits, but by intrigues and by the empiricism which has invaded the whole civilized world.

Having thus spoken, Leonard asks himself: "What is cholera?" Upon this question is what he has been meditating in his solitude in the country since 1854. At last he has it. "*It is nothing more than a consequence of the displacement of the worms of which we are made up.* A sudden fear, an indigestion, or any other disorder which shakes but does not kill the delicate mass of worms, will arouse them, and they, leaving their normal place, will assail and suffocate the patient, as frequently happens in nursing children. The rice pap discharges are nothing else but a mass of smaller worms with their excreta, triturated by the current of the larger worms which assail the belly, stomach and throat. The greater or less intensity of the assault are proportionate to the greater or less intensity of the disease, and hence the *cholerine*, the *cholera*, and the *cholerone*"

After having reproached physicians, who were not willing to risk their lives by making any autopsy on the cadavers, for the disease is highly contagious, and adding that it is "one thing to speak about death and courage, and another is surely to die," he gives a very interesting explanation why cholera becomes diffued and contagious. "Because the worms, offended by the stench of the cavaders of their brethren, try to escape from their place, and in so doing violently assail the belly, stomach, and throat, causing the choleraic symptoms, and also death, according to the intensity of the assault, as already described above."

Leonard, not yet content with having done so much good for humanity, sacrifices all himself . . . . . . and suggests remedies indicated to pacify and replace the worms, and to kill those who are enraged when they threaten our lives with deadly assaults, viz.: For cholera and cholerine, strong coffee, olive oil, rum, bitter decoctions, etc. For the *cholerone* . . . . . . according to the strength of the patient." These points constitute his secret nostrum, *discovered by chance* and *much tried*, which he will make known when "asked by the authorities." He is also "willing to do this if public opinion will favorably receive and appreciate his bold publication."

Alas, Leonard, truly enough thou shalt have to bury thy secret! Ungrateful society, instead of appreciating thy bold publication, shows thee the way to the insane asylum!

A. LAGORIO, M. D.

Chiavari, Dec. 1st, 1884.

